# Language use on Twitter reflects social structure and social disparities

**DOI:** 10.1016/j.heliyon.2023.e23528

**Published:** 2023-12-12

**Authors:** Eric Mayor, Lucas M. Bietti

**Affiliations:** a University of Basel, Switzerland; b Norwegian University of Science and Technology, Norway

**Keywords:** Mental health, Area deprivation index, Ethnicity, LIWC, Twitter

## Abstract

Large-scale mental health assessments increasingly rely upon user-contributed social media data. It is widely known that mental health and well-being are affected by minority group membership and social disparity. But do these factors manifest in the language use of social media users? We elucidate this question using spatial lag regressions. We examined the county-level (*N* = 1069) associations of lexical indicators linked to well-being and mental health, notably depression (e.g., first-person singular pronouns, negative emotions) with markers of social disparity (e.g., the Area Deprivation Index–3) and ethnicity, using a sample of approximately 30 million content-coded tweets (U.S. county-level aggregation). Results confirmed most expected associations: County-level lexical indicators of depression are positively linked with county-level area disparity (e.g., economic hardship and inequity) and percentage of ethnic minority groups. Predictive validity checks show that lexical indicators are related to future health and mental health outcomes. Lexical indicators of depression and adjustment coded from tweets aggregated at the county level could play a crucial role in prioritizing public health campaigns, particularly in socially deprived counties.

## 1 Introduction

Assessing public health through surveys and interviews is time-consuming and expensive [1]. The analysis of social media messages provides a more sustainable alternative, enabling swift, large-scale assessments of mental health and well-being [[1], [2], [3], [4], [5], [6], [7]]. Despite the non-representativeness of Twitter users in comparison with the general population [8], several studies report excellent convergence of such indicators with self-reported measures (e.g. Ref. [9]), including at the county level [10]. This demonstrates that social media data are “good enough” for the estimation of well-being at the population level.

The relationship between language use and mental health has been studied using the Linguistic Inquiry Word Count (see Ref. [11]), more recently using social media messages as data (e.g. Ref. [12]). Further, research has shown that language in social media messages can accurately predict a future depression diagnosis [13]. Two main approaches are generally used for the study of well-being and mental health, relying upon textual production such as social media posts or personal narratives: the open-vocabulary approach and the closed-vocabulary approach [14]. The open-vocabulary approach uses methods specialized in extracting meaning from the data in a bottom-up fashion, such as machine learning and latent Dirichlet allocation (e.g. Ref. [15]). While the reliance on machine learning has proven a useful approach to the assessment of mental health using social media data on a large scale (e.g. Ref. [16]), such tools decrease in performance over time due to data or model drift (e.g. Ref. [17]). Such models, therefore, require frequent updates, a process known to be error-prone, and requiring frequent revalidation under best practices, which requires continued access to so-called *labelled data*, i.e., data for which the outcome is also known (e.g. Ref. [18]). Such complexity and associated costs might be worthwhile given the high societal importance of mental health and well-being. But there is also room in research and practice for less efficient but more understandable (in the sense of linear relationships between known variables) and less costly approaches, such as the use of well-researched closed vocabularies.

The closed-vocabulary approach uses lexica that were defined and validated prior to data collection. Most studies on social media and well-being relying upon a closed vocabulary use the Linguistic Inquiry Word Count (LIWC) [11]. The LIWC is a software package for which several versions were created over the years, starting with LIWC2001 [19] with continuously improved categories, enabling the automated coding of dozens of indicators from text [20].

The percentages of pronouns and emotional words as coded by the LIWC have long been identified as indicators of well-being in personal narratives (e.g. Ref. [21]). What is meant by the term ‘indicator’ is that the identified lexical variable reflects shared variance with well-being, generally as self-reported by study participants. The use of the term ‘indicator’ does not imply knowledge of a causal direction, even in the case of a temporal association. Pronouns are an indicator of the target of attention of the speaker or writer [11,22]. The focus on the self, with the use of first-person singular pronouns such as I, mine, etc., has been frequently linked with depressive symptomatology (e.g. Refs. [12,21,23,24]). Other lexical categories coded by the LIWC are associated with depressive symptoms: negative emotions (positively [7,12,13,21,24,25]); positive emotions (negatively [21,[24], [25], [26]]), social words (negatively [21]), second person pronouns (positively [12,24]; but also negatively [27]), swear words (positively [24]), anger (positively [7]), and sadness (positively [7,13]). Most of these studies were conducted with data collected on social media [12,13,[24], [25], [26]]. [28] notably find negative emotions, first-person singular pronouns, anger, and third-person pronouns to be negatively related to well-being and positive emotions, and first-person plural pronouns to be positively related to well-being in tweets (county-level associations).

A recent publication [29] shows positive emotions (negatively) and negative emotions (positively) to be related to the diagnosis of depression (among other LIWC-2015 dimensions). We note that contrary to temporal-level and individual-level studies such as [9,16,29] have found the opposite association of the positive emotions category of the LIWC (and other similar categories of other instruments) with subjective well-being at the county level.

### 1.1 This study

Mental health and well-being are robustly affected by social deprivation both at the individual level and county level (e.g., living in poverty, having lower occupational status or lower education attainment, and being unemployed; e.g., Refs. [[30], [31], [32]]).

African Americans and Hispanics exhibit significantly higher levels of depressive symptoms compared to White individuals in the U.S. [33,34], but see Ref. [35]. Latinos have a higher likelihood of meeting depressive disorder criteria compared to Whites and have higher levels of chronic stress and more unhealthy behavior [36]. While acute depression (meaning an increase in depressive symptomatology in the two weeks prior to an assessment in comparison to before) is less frequently observed in African Americans compared to Whites, African Americans more frequently suffer from prolonged depressive symptoms than Whites, i.e., chronic depression [33]. Mental health disparities were also present during the COVID-19 pandemic, as high levels of psychological distress were observed in 22 % of Latinos, 18 % of Asians, 16 % of African Americans, and 14 % of Whites [37]. Further, the mental health of ethnic minorities decreased more during the pandemic in comparison to Whites [38]. We note that higher rates of depressive symptomatology among ethnic minority members can largely be attributed to discrimination and prejudice, lower socioeconomic status, higher exposure to stressors, health burdens, and a lack of health insurance [33,39] and that racial and ethnic differences in depressive symptomatology could be partially attributed to differences in family background and wealth [34].

Previous studies have shown that county-level indicators, such as the proportion of ethnic minority members, are associated with language use on Twitter [40] and that Twitter language converges with public health county-level physical and mental health estimates [10,41]. Despite these accomplishments, whether social deprivation and ethnic minority membership status are reflected in large-scale language use associated with depression and adjustment on Twitter remains, to date, an unanswered question. This question, which we seek to answer in this contribution, falls in the purview of geographic psychology, which is interested in the study of the differences in behavior, personality, attitudes, and emotion/well-being across regions as well as their correlates [42,43]. The construct “ecological fallacy” (see Ref. [44]) relates to the issue of supposing relationships between variables measured at the level of the individual are also present at a higher level. In this contribution, we have verified that relationships that were observed in past literature of lexical indicators with mental health and well-being at the individual level do hold in most instances at the aggregate level (counties) (see also [10]). Lexical indicators of particular relevance for such an inquiry are those known to be linked with mental health and depression notably (e.g. Refs. [11,12,24]). In the context of this study, for brevity, we refer to indicators positively associated with depressive symptomatology as *indicators of depression* and to indicators negatively associated with depression as *indicators of adjustment*.

Here, we examine the association of such linguistic indicators coded from 30 million geolocated tweets from 1069 U S. counties (county-level aggregation) with county-level markers of social structure and social disparity (area deprivation) [45]. We expected that the proportion of ethnic minority status individuals and county-level area deprivation would be positively associated with lexical indicators of depression (e.g., negative emotions) and negatively associated with indicators typically negatively linked to depression (e.g., positive emotions, see above and Materials and Methods).

We test these associations relying upon spatial lag regression as this allows accounting for the spatial dependence of observations, which could otherwise bias the results. This also affords the opportunity to disentangle direct and indirect effects from the total effects of the predictors [46], which is relevant when examining spatial data in general. In our case, this answers the additional research question: Is language use in counties only linked with county characteristics within the same county, or is it also associated with the characteristics of nearby counties?

## 2 Materials and Methods

*Twitter sample and pre-processing.* In October and November 2018 (from October 3rd to November 26th), we collected 31.8 million English-language tweets using the Twitter streaming API with R package *rtweet*. Tweets were geo-located within the bounding box of the U.S. (defined as {-125, 26, −65, 49}). We preprocessed the tweets by removing URLs, mentions, non-ascii characters, punctuation, and digits. Contractions were replaced with long-form content using the textclean package [47].

*County localization.* We were able to assign 30.4 million tweets from 1.85 million users (on average 17.17 tweets per user, SD = 81) to a U.S. county based on the obtained coordinates information relying upon R packages *sp* [48]*, maps* [49], and *maptools* [50]. The total number of tweets per county was, on average, 10,897 (SD = 49,131). The average number of tweets per county and date was 248.3 (SD = 1012) (see detailed table of frequencies and the script assigning tweets to counties on OSF.io). The number of U.S. counties with at least one tweet assigned was 2794 out of 3232. For further analyses, we included only counties for which we had sufficient data to obtain aggregated estimates of our dependent variables (at least 20 tweets per day on 35 or more days; see below).

*Coded variables from tweets (criterion variables)*. We coded each tweet using the LIWC-2015 dictionary [20]. From these, we selected several categories with documented associations with depression [24].

Indicators of depression.
  - negative emotions (e.g., *bad*, *hurt*; positive association with depressive symptoms). We also included (see tables in Supplementary information file 1) the discrete emotion categories anger, sadness, and anxiety – which were associated with depressive symptomatology in past research; e.g. Ref. [51]);
  - first-person singular pronouns (e.g., *me, I, mine*; positive association), second-person pronouns (e.g., *you, your*; positive association);
  - swear words (e.g., *damn*; positive association);
  - negations (e.g., *not, never*; positive association).

Indicators of adjustment.
  - first-person plural pronouns (*we, our*; negative association with depression). We also included the ratio of such pronouns to the total of first-person pronouns in order to ascertain that results are not simply a matter of higher pronoun use in some counties versus others;
  - articles (e.g., *a, an, the*; negative association with depression);
  - positive emotions (e.g., *good, love*; negative association with depression).

*Data aggregation.* We selected the tweets that were from a county for which we had at least 20 tweets per day on 35 or more days during the data collection period (1069 counties). A selection based on the minimal number or frequency of observations is commonplace [46]. We determined our criteria prior to data analysis: In order to state that the assessed variables related to well-being over the close to 60-day period of data collection, we decided that more than half of the total days should be included. We also considered a value of 50 tweets per day. However, this value rendered the number of included counties after selection based on the same minimum number of days to drop to around 750 counties, which was deemed too few, while a value of 10 tweets per day seemed too small. In other words, the selection criteria we used allow for addressing both the generalizability of the results and the robustness of the estimates. Fig. S1 (Supplementary information file 1; computed after the choice of the “20 tweets for 35 days or more” selection criteria) shows the correlations do not change appreciably depending on the tested criteria for county selection (except when all counties are included – lower associations).

We aggregated the data at the user level first, then at the county level to reduce the influence of accounts that tweeted disproportionately on county-averaged values (see Ref. [52]). The measurements are more precise for some of the counties compared with others. The measurement for the county of Dickson (TN, 2013–2017 population according to the American Community Survey – ACS: 51,341) is the average of the values for 875 tweets, while the measurement for Los Angeles (CA, 2013–2017 population according to the ACS: 10, 105, 722) is the average of close to 1.5 million tweets.

*Publicly available county-level variables (predictors)*. We used publicly available data at the county-level, collected through the U.S. Census Bureau's Application Programming Interface (API). We used R function get_acs() of the tidycensus package to obtain or compute the ACS 2013–2017 five-year estimates of sociodemographic data (percent male, median age, percent White – race alone or in combination with one or more other races, percent Hispanic or Latino, percent Black or Afro-American, percent Asian). We used the function get_adi() of the sociome package to obtain data for the revised Area Deprivation Index as well as its 3 dimensions (Financial strength and Educational attainment – positive indicators of deprivation, as well as Economic hardship and inequality – negative indicator of deprivation [45]). We also included in the analyses the following indicators, which were returned by the function call, as these are components (among others) of the ADI and its dimensions: percent with less than ninth-grade education, percent with at least a high school education, percent unemployed, and median family income.

*Publicly available county-level health and mental health variables (predictive validity).* We obtained data from the County Health Rankings 2022 which collates previous data for different surveys (about the County Health Rankings, see Ref. [53]). Among variables included in the dataset, we used the proportion of poor physical health days and of poor mental health days from 2019 to check that our selection of lexical categories was indeed associated with relevant public health measures.

*Data analysis.* «[S]patial data often violate the assumptions of statistical techniques commonly used by psychologists. Specifically, a basic assumption in regression analysis is that observations should be independent and statistical dependence of observations can lead to estimation biases» ([46], p.2). This issue can be addressed using spatial analyses [54]. Adapting code from Ref. [46], we relied upon spatial lag models to regress our dependent variables on our independent variables individually using the package *spdep* in R [55], each time controlling for percent male, median age, and population density. We note 88 regions were not adjacent to any other region with data in our sample of tweets and were automatically excluded from the analyses. In Supplementary information file 2, we include the estimates of direct, indirect, and total effects for all variables included in the models (values for the control variables are omitted from the tables in the manuscript). We estimated residual autocorrelation using the Moran's I statistic [56] and its deviate, provided in Supplementary Table S1 (Supplementary information file 1) for our main analyses and Table 4 for the predictive validity checks. Moran's I values have a theoretical range of −1 to 1 (with 0 indicating no clustering). In this case, they refer to the degree of clustering of the regression residuals. The Moran's I deviate values are z-scores which relate the observed value to an expected value under a random distribution. The county-level aggregated data and script for analyses are available on the Open Science Framework platform at: https://osf.io/t2ew7.

In the main text, we present the beta coefficients for the total effects, excluding for legibility reasons anxiety, anger, and sadness. Tables S2–S4 (in Supplementary information file 1) also include these variables as well as the direct and indirect effects, which were, in most instances, both significant when the total effect was significant.

## 3 Results

**Fig. 1:**
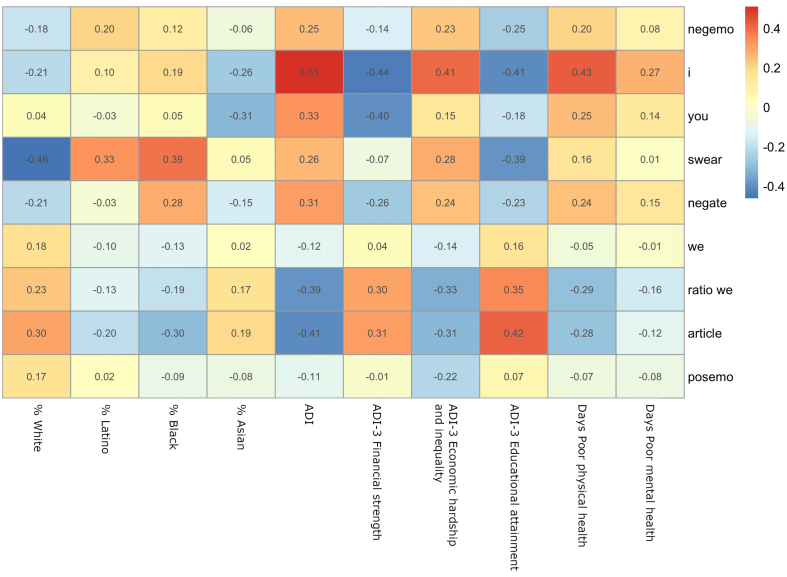
Zero-order correlations of the county-level sociodemographic variables, the Area Deprivation Index Variables, and days of poor days of physical health and days of poor mental health with selected LIWC-2015 indicators of depression and of adjustment. *Note*. Negemo: negative emotions; i: first-person singular pronouns; you: second-person pronouns; swear: swear words; negate: negations; we: person plural pronouns; ratio we: ratio of first-person plural pronouns over all first-person pronouns; posemo: positive emotions; Correlations with absolute values above 0.06 are significant at *p* < .05.

In Fig. 1, we present zero-order correlations of the county-level sociodemographic variables, the Area Deprivation Index Variables, and poor days of physical health and mental health with selected LIWC-2015 indicators of depression and indicators of adjustment. As mentioned above, we excluded anxiety, anger, and sadness from the manuscript tables (but see Supplementary information file 1). The negative emotions category includes these discrete emotions. Anxiety was generally not associated with the independent variables, whereas depression and anger, in most instances, had associations very similar to the supraordinate lexical category negative emotions.

The correlations show that our lexical variables are generally associated with sociodemographic variables, the ADI, and the ADI-3 dimensions in the expected direction, except for associations with the percentage of Asian residents. Further, our main lexical variables are correlated in the expected direction with either or both poor mental health days and poor physical health days, with the exception of first-person plural pronouns for which correlations are often non-significant (contrary to the ratio of first-person plural pronouns). The correlations between sadness, anxiety, and anger with the ADI can be seen in Supplementary Fig. S1 (Supplementary information file 1).^1^

The Moran I values for the regression residuals and their deviate are displayed in Supplementary Table S1 (see Supplementary information file 1). None of the values reached significance according to the moran.test() function output, yet one Moran's I value had a deviate below −1.96 (the model with first-person singular pronouns as DV and ADI-3 Economic hardship and inequality as IV). As Moran's I are z-scores, this indicates that for that model, the residuals of geographical units that are spatially close are more dissimilar than expected.

*County-level associations of indicators of depression and adjustment with ethnicity.* We comment on the results relating to the total effect of the independent variables below. We expected the proportion of ethnic minority residents to be positively associated with lexical indicators of depression (e.g., negative emotions, first-person singular pronouns) and negatively with lexical indicators of adjustment (e.g., positive emotions, first-person plural pronouns). We expected the opposite associations for the proportion of White residents. For most cases, this assumption was supported (see Table 1 and Supplementary Table S2 in the Supplementary information file 1 for the complete list of variables and direct, indirect, and total effects): The percentage of White residents was negatively associated with negative emotions, anger, first-person singular pronouns, swear words, and negations. It was positively associated with sadness (contrary to expectations), first-person plural pronouns, the ratio of first-person plural pronouns, articles, and positive emotions. The percentage of Latino residents was negatively associated with first-person plural pronouns, ratio of first-person plural pronouns, and articles. It was positively associated with negative emotions, anger, sadness, first-person singular pronouns, and swear words. The percentage of African American residents was negatively associated with sadness (contrary to expectations), first-person plural pronouns, the ratio of first-person plural pronouns, articles, and positive emotions. It was positively associated with negative emotions, anger, first-person singular pronouns, swear words, and negations. The percentage of Asian residents was negatively associated with negative emotions, sadness, first-person singular pronouns, second-person pronouns, and negations (all contrary to expectations). It was positively associated with the ratio of first-person plural pronouns and articles (both contrary to expectations).

**Table 1:** Spatial lag regression total effects Betas for percentage of different ethnic groups as independent variables (controlling for population density, proportion of males, and median age).

	% Asian	% African American	% Latino	% White
negemo	−0.11 *	0.19 ***	0.32 ***	−0.21 ***
i	−0.37 ***	0.33 ***	0.26 ***	−0.27 ***
you	−0.31 ***	0.07	0.01	0.04
swear	−0.07	0.60 ***	0.41 ***	−0.63 ***
negate	−0.19 ***	0.32 ***	0.03	−0.24 ***
we	−0.02	−0.15 ***	−0.13 ***	0.20 ***
ratio we	0.18 ***	−0.28 ***	−0.21 ***	0.29 ***
article	0.22 ***	−0.47 ***	−0.26 ***	0.45 ***
posemo	0.01	−0.17 ***	0.04	0.18 ***

*Note.* Indicators of depression: negemo, i, you, swear, negate; Indicators of adjustment: we, ratio we, article, posemo. Negemo: negative emotions; anx: anxiety; sad: sadness; i: first-person singular pronouns, you: second-person pronouns; swear: swear words; negate: negations; we: person plural pronouns; ratio we: ratio of first-person plural pronouns over all first-person pronouns; posemo: positive emotions. *: *p* < .05, **: *p* < .01; ***: *p* > .001.

In summary, as indicated in Table 1, results in most instances support our expectations regarding the associations of ethnicity and lexical indicators of depression and adjustment. The main exception to this is the sociodemographic variable percent Asian with associations which generally were the reverse of those expected. Overall, the profile of associations of the percentage of Asian residents resembled more that of the percentage of White residents than of Latino or Black residents.

### 3.1 Disparity

We first present results relating to the ADI and its dimensions. We then continue with some of their components.

*County-level associations of indicators of depression and adjustment with the ADI and its dimensions.* We expected lexical indicators of depression to be positively associated with the Area Deprivation Index (ADI) and the ADI-3 dimension of Economic hardship and inequality [45]. We expected negative associations of the ADI-3 dimensions of Financial strength and Educational attainment [45] with lexical indicators of depression. We expected the opposite relationships for lexical indicators of adjustment. Table 2 presents these results (also see Supplementary Table S3).

**Table 2:** Spatial lag regression total effects Betas for the ADI and the ADI-3 dimensions as independent variables (controlling for population density, proportion of males, and median age).

	ADI	ADI-3 EHI	ADI-3 EA	ADI-3 FS
negemo	0.28 ***	0.26 ***	−0.34 ***	−0.20 ***
i	0.60 ***	0.54 ***	−0.46 ***	−0.57 ***
you	0.29 ***	0.12 **	−0.12 **	−0.40 ***
swear	0.39 ***	0.42 ***	−0.49 ***	−0.22 ***
negate	0.27 ***	0.22 ***	−0.18 ***	−0.27 ***
we	−0.08 *	−0.11 **	0.12 **	0.02
ratio we	−0.42 ***	−0.39 ***	0.33 ***	0.37 ***
article	−0.43 ***	−0.38 ***	0.42 ***	0.35 ***
posemo	−0.13 ***	−0.23 ***	0.08 *	0.04

*Note*. Indicators of depression: negemo, i, you, swear, negate; Indicators of adjustment: we, ratio we, article, posemo. Negemo: negative emotions; anx: anxiety; sad: sadness; i: first-person singular pronouns; you: second person pronouns; swear: swear words; negate: negations; we: person plural pronouns; ratio we: ratio of first-person plural pronouns over all first-person pronouns; posemo: positive emotions; ADI: Area Deprivation Index; EHI: Economic hardship and inequality; EA: Educational attainment; FS: Financial strength. *: *p* < .05, **: *p* < .01; ***: *p* > .001.

The ADI was negatively associated with first-person plural pronouns, the ratio of first-person plural pronouns, articles, and positive emotions. It was positively associated with negative emotions, anger, sadness, first-person singular pronouns, second-person pronouns, swear words, and negations. The ADI-3 Financial strength dimension was negatively associated with negative emotions, anger, sadness, first-person singular pronouns, second-person pronouns, swear words, and negations. It was positively associated with the ratio of first-person plural pronouns and articles. The ADI-3 Economic hardship and inequality dimension was negatively associated with first-person plural pronouns, the ratio of first-person plural pronouns, articles, and positive emotions. It was positively associated with negative emotions, anger, sadness, first-person singular pronouns, second-person pronouns, swear words, and negations. The ADI-3 Educational attainment dimension was negatively associated with negative emotions, anger, sadness, first-person singular pronouns, second-person pronouns, swear words, and negations. It was positively associated with first-person plural pronouns, the ratio of first-person plural pronouns, articles, and positive emotions.

In summary, in the large majority of instances, the results corroborated our expectations regarding the associations of the structural indicators of the ADI and ADI-3 and the linguistic indicators of depression and adjustment.

County-level associations of indicators of depression and adjustment with selected components of the ADI and ADI-3.

As an additional analysis, we examined whether lexical indicators of depression were positively associated with the following components of the ADI and ADI-3: percent with less than ninth-grade education, percentage of residents with at least a high school education, percentage unemployed, and median family income (Table 3, also see Supplementary Table S4). We expected the opposite relationships for lexical indicators of adjustment. Income, education, and unemployment were suggested by reviewers as control variables, but as these are components of the ADI (and of the ADI-3 dimensions), we rather included those analyses separately (i.e., it would be difficult to interpret the effect of education attainment controlling for percent with at least a high school education).

**Table 3:** Spatial lag regression total effects Betas for percent of residents with less than ninth-grade education, at least a high school education, unemployed as well as median family income as independent variables (controlling for population density, proportion of males, and median age).

	% < Ninth-grade edu.	% ≥ HS edu.	% unemployed	Median fam. income
negemo	0.29 ***	−0.35 ***	0.36 ***	−0.22 ***
i	0.39 ***	−0.55 ***	0.50 ***	−0.56 ***
you	0.09	−0.21 ***	0.07	−0.29 ***
swear	0.42 ***	−0.51 ***	0.54 ***	−0.29 ***
negate	0.15 ***	−0.27 ***	0.18 ***	−0.24 ***
we	−0.10 **	0.13 ***	−0.16 ***	0.04
ratio we	−0.27 ***	0.39 ***	−0.39 ***	0.38 ***
article	−0.37 ***	0.48 ***	−0.38 ***	0.37 ***
posemo	−0.05	0.09 *	−0.20 ***	0.12 **

*Note.* Indicators of depression: negemo, i, you, swear, negate; Indicators of adjustment: we, ratio we, article, posemo. Negemo: negative emotions; anx: anxiety; sad: sadness; i: first-person singular pronouns; you: second-person pronouns; swear: swear words; negate: negations; we: person plural pronouns; ratio we: ratio of first-person plural pronouns over all first-person pronouns; posemo: positive emotions; < ninth-grade educ.: less than ninth-grade education; ≥ HS educ.: at least a high school education; Median fam. income: Median family income. *: *p* < .05, **: *p* < .01; ***: *p* > .001.

**Table 4:** Spatial lag regression Betas for the number of poor physical and mental health days regressed on the LIWC-2015 indicators of depression and adjustment.

	Days of poor physical health (DV)	Days of poor mental health (DV)
negemo	0.28 ***	0.20 ***
i	0.58 ***	0.49 ***
you	0.25 ***	0.19 ***
swear	0.24 ***	0.11 *
negate	0.32 ***	0.23 ***
we	−0.04	−0.02
ratio we	−0.39 ***	−0.33 ***
article	−0.35 ***	−0.24 ***
posemo	−0.17 **	−0.15 **

*Note*. Indicators of depression: negemo, i, you, swear, negate; Indicators of adjustment: we, ratio we, article, posemo. Negemo: negative emotions; anx: anxiety; sad: sadness; i: first-person singular pronouns; you: second-person pronouns; swear: swear words; negate: negations; we: person plural pronouns; ratio we: ratio of first-person plural pronouns over all first-person pronouns; posemo: positive emotions. *: *p* < .05, **: *p* < .01; ***: *p* > .001.

The percentage of residents with less than ninth-grade education was negatively associated with first-person plural pronouns, the ratio of first-person plural pronouns, and articles. It was positively associated with negative emotions, anger, sadness, first-person singular pronouns, swear words, and negations. The percentage of residents with at least a high school education was negatively associated with negative emotions, anger, sadness, first-person singular pronouns, second-person pronouns, swear words, and negations. It was positively associated with first-person plural pronouns, the ratio of first-person plural pronouns, articles, and positive emotions.

Median family income was negatively associated with negative emotions, anger, sadness, first-person singular pronouns, second-person pronouns, swear words, and negations. It was positively associated with the ratio of first-person plural pronouns, articles, and positive emotions. The percentage of unemployed residents was negatively associated with first-person plural pronouns, ratio of first-person plural pronouns, articles, and positive emotions. It was positively associated with negative emotions, anger, sadness, first-person singular pronouns, swear words, and negations. All other associations involving our independent variables are non-significant. The associations, including the control variables for the different models, are available in the complete outputs in Table 3.

### 3.2 Visualizations of indicators of depression and sociodemographic and structural indicators

**Fig. 2:**
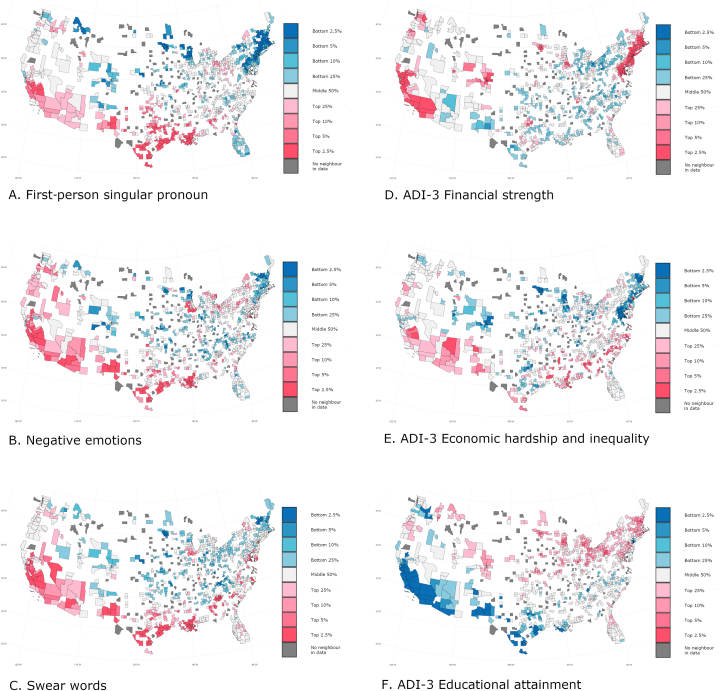
County-level visualizations of several lexical variables and the 3 dimensions of the ADI-3 accounting for spatial clustering (Getis-Ord Gi scores).

Adapting code from Ref. [46], we relied upon Getis-Org Gi [57] with the spdep R package [55] to plot hotspots and coldspots on the variables described below accounting for spatial clustering. On the left side, Fig. 2 includes county-level visualizations of the ranking of several lexical variables. On the right side, it presents the 3 dimensions of the ADI-3 which illustrate the similarity of the ranking of the lexical indicators of depression across counties with the ranking of the area deprivation variables.

### 3.3 Predictive validity checks

In order to verify that our selection of lexical categories was indeed associated with relevant public health measures, we computed spatial lag models with the proportion of poor physical and mental health days in 2019 as dependent variables regressed on each of the lexical indicators (with the following control variables each time: population density, median age, and percent of male residents). These analyses, of which the results are presented in Tables 4 and Table S5, thus serve as an indicator of predictive validity. Both poor physical health days and poor mental health days were positively associated with all indicators of depression (except anxiety; see Table S5). Both poor physical health days and poor mental health days were negatively associated with all indicators of adjustment except first-person plural pronouns.

### 3.4 Direct, indirect, and total effects

The interpretation of the results above relates to the total effects (how much the independent variables affect the dependent variables overall). An examination of the direct and indirect effects (see Supplementary information file 1) allows us to answer our additional research question: Is language use in counties only linked with county characteristics within the same county (significant direct and total effects), or is it also associated with the characteristics of nearby counties (significant indirect effects)? We observe (Tables S2–S5) that in almost all cases of significant total effects, both the direct (effect of the increase of the IV within the same county) and the indirect effect (effect of the increase of the IV in adjacent counties) are significant, meaning that the association of the independent variables in a county with lexical indicators of depression and adjustment is not limited to the same county, but extends to nearby counties.

### 3.5 Synthesis

We performed spatial lag analyses in order to ensure that the associations between predictors and outcome variables were not biased by spatial autocorrelation (e.g. Ref. [46]). The non-significance of the Moran's I values on the residuals of our models shows that the degree of spatial autocorrelation is not different from what is expected by random county allocation. The results of the spatial lag regression show good convergence with the correlations shown in Fig. 1 (main text) and S1 (Supplementary information file 2). We expected the proportion of minority residents to be negatively associated with lexical indicators of adjustment. Conversely, we expected the opposite associations for the proportion of White residents. In most cases, this assumption was supported. We anticipated lexical indicators of depression to be positively associated with the ADI and the ADI-3 dimension of Economic hardship and inequality (and component variables percent with at least a high school education and median family income). We expected negative associations of the ADI-3 dimensions of Financial strength and Educational attainment (and component variables percent with less than a high school education as well as percent unemployed) with lexical indicators of depression. We expected the opposed relationships for lexical indicators of adjustment. These expectations were largely confirmed in the majority of instances, as the results showed associations between the structural indicators of the ADI, the ADI-3, and their component variables with the linguistic indicators of depression and adjustment.

## 4 Discussion

We sought to investigate whether large-scale language use on Twitter reflects social structure and social disparities. We have analyzed the data using spatial lag regression in order to ensure that the reported associations were not accounted for by the spatial dependence of the observations. In most instances, the proportion of ethnic minority residents was positively associated with lexical indicators of depression and negatively associated with lexical indicators of adjustment. Such results have confirmed our expectations (exceptions: percentage Asian (IV), anxiety (DV)).

We have thus shown that, indeed, county-level area deprivation and proportion of minority groups were associated, sometimes strongly, with linguistic indicators of depression and adjustment. We have further assessed the predictive validity of the linguistic indicators of depression and adjustment. Results show that most indicators, indeed, related to future poor physical and mental health days in the 1069 U.S. counties included in the study. Interestingly, the effects of the independent variables cross county boundaries, as can be observed from the significance of the large majority of indirect effects in the spatial lag models. Such analyses allow the identification and quantification of spillover effects, which are crucial for decision-making in public health policies and campaigns [58]. For instance, in the case of our study, county-level area deprivation was not only associated with same-county indicators of depression and adjustment but also with such indicators of well-being in other counties.

The goal of this study is not to supplement current public health statistics (others have, better than we did, shown the convergence of these with social media data, e.g. Ref. [10]), but to test specific hypotheses and highlight the value of social media data, which provide researchers with the unprecedented opportunity to assess geographic variations in lexical indicators of depression and relate these to area deprivation at population levels. Our study represents a unique source of information for the prioritization of public health campaigns, particularly in regions featuring lower overall socioeconomic status and higher ethnic minority representation. Collecting such data can be accomplished almost in real time and lexical changes within regions could anticipate large-scale forthcoming changes in public health. Under-reporting of mental health symptoms is related to ethnicity and socioeconomic status notably (e.g. Ref. [59]). This is of utmost importance considering that members of socially deprived communities might therefore be the most at risk but the least likely to seek care. For example [60], reported that black participants with clinically significant depressive symptoms and/or diagnosed depression were 61 % less likely to report receiving any treatment (medications and/or counseling) compared to non-Hispanic White participants. Mental illness stigma has been found to be a more significant factor limiting help-seeking behaviors among ethnic minorities [61] than among Whites. This highlights the importance of targeted public health campaigns. Ethnic minorities living in socially deprived areas tend to show more severe and persistent symptomatology with higher levels of impairment than non-Hispanic Whites [62,63]. Estimating changes in the use of language in terms of indicators of depression and adjustment at the county level can orient public health interventions towards the counties that need them most at the time. For instance, a county in which social media posts show an increased use of lexical indicators of depression over a relatively long period of time (e.g., one month) might be more in need of a community intervention than a county with a similar area of social deprivation but instead an increase in the use of lexical indicators of adjustment for the same period.

### 4.1 Strengths

Using a sample of dozens of millions of Tweets from more than 1 million users, we have shown that the use of language indicative of depression and well-being on Twitter reflects social deprivation and ethnic minority membership. Relying upon language use, as we did in this study, allows for estimating changes in depressive symptomatology and well-being at a fine-grained spatial and temporal resolution at a large scale [5,52]. We have relied upon spatial lag regressions to test our hypotheses. This is a strength of this study, as it allows us to ensure that biases due to spatial dependence (e.g. Ref. [54]) are absent from the reported associations, thereby enhancing model fit and predictive precision [46]. We note depressive symptomatology can be under-reported by ethnic minority members, including in public health surveys [59]. The non-obtrusiveness of social media data is also an advantage. In a tutorial. [64] have detailed the use of signal analysis methods for regularly sampled coded text. Such use of these tools could be useful for public health purposes, for instance to detect change points in linguistic indicators of depression and wellbeing.

### 4.2 Limitations

In this study, we have examined county-level associations of lexical indicators of depression and well-being with sociodemographic and area deprivation variables on the one hand as well as physical and mental health on the other. The ADI and the dimensions of the ADI-3 are only one of many ways to operationalize area deprivation: There are other operationalizations (e.g., we replicated our findings using the Social Deprivation Index of [65], see Supplementary information file 2). Another limitation relates to the need for a selection of counties for the study. The modifiable aerial problem [66] is also a limitation of this study, namely, how the magnitude of results might depend upon the level of data aggregation. Because we didn't have enough data for all the counties, we couldn't further aggregate at the state level and examine whether the magnitude of our results augmented at that level of aggregation. Regarding the LIWC-2015 positive affect dimension, [16] noted that regional word use could reverse the associations of the overall dimension. While this was not the case in our study—we generally found small positive associations in the expected direction—our results for this category might have been stronger after the exclusion of the problematic words mentioned by Ref. [16] – but this would have made comparisons with other studies using LIWC-2015 difficult.

## 5 Conclusion

In this study, we showed that in a sample of approximately 30 million content-coded tweets (U.S. county-level aggregation), language use reflected social structure, with ethnic minority individuals showing higher indicators of depression and lower indicators of adjustment. We also found that county-level area deprivation was positively associated with lexical indicators of depression and negatively associated with indicators of adjustment. Social media data can be useful for assessing mental health trends through language use, particularly at periods or durations for which no public health data is available (e.g., comparing one week to the other or estimating the impact of dramatic events).

## Data availability

The data has been uploaded on osf. io. Reviewers can access the data here: https://osf.io/t2ew7.

## CRediT authorship contribution statement

**Eric Mayor:** Writing – review & editing, Writing – original draft, Visualization, Validation, Project administration, Methodology, Investigation, Formal analysis, Data curation, Conceptualization. **Lucas M. Bietti:** Writing – review & editing, Writing – original draft, Conceptualization.

## Declaration of competing interest

The authors declare that they have no known competing financial interests or personal relationships that could have appeared to influence the work reported in this paper.

## Footnotes

1 Fig. S1 in the Supplementary information file 1 presents the correlations of the ADI, the LIWC-2015 negative emotions category, and the LIWC-2015 first-person pronoun category with all study variables using different county inclusion criteria. The figure shows that the correlations are close to identical for all the examined inclusion criteria and that the correlations of the mentioned LIWC-2015 categories are somewhat lower, relying upon the complete dataset, which is not the case for the correlations of the ADI.
