# Supplementary material for: Language use on Twitter reflects social structure and social disparities
Source: Heliyon. 2023 Dec 12;10(2):e23528. doi: 10.1016/j.heliyon.2023.e23528 (PMC10825303; doi:10.1016/j.heliyon.2023.e23528)
Supplement: Multimedia component 1 [file mmc1.docx]

Supplementary materials file 1 to:

Language use on Twitter reflects social structure and social disparities

Eric Mayor 1 and Lucas Bietti 2

1 University of Basel, Switzerland

2 Norwegian University of Science and Technology, Norway

Corresponding author: * Eric Mayor

**Email:** [ericmarcel.mayor@unibas.ch](mailto:ericmarcel.mayor@unibas.ch)

Table S1. Moran’s I statistic (Moran’s I statistic deviate) for the main models

|  | % Asian | | % Afro-American | % Latino | % White | ADI | ADI-3 EHI | ADI-3 EA | ADI-3 FS | % < 9th grade educ. | % >= HS educ. | % unempl. | Med. fam. income |
| --- | --- | --- | --- | --- | --- | --- | --- | --- | --- | --- | --- | --- | --- |
| negemo | | -0.010  (-0.33) | -0.019  (-0.61) | 0.000  (0.04) | -0.021  (-0.71) | -0.015  (-0.49) | -0.024  (-0.82) | -0.013  (-0.43) | -0.008  (-0.24) | -0.016  (-0.51) | -0.017  (-0.55) | -0.023  (-0.76) | -0.011  (-0.35) |
| anx | | 0.000  (0.04) | -0.001  (0.01) | 0.000  (0.04) | 0.000  (0.02) | 0.001  (0.06) | 0.001  (0.05) | 0.000  (0.04) | 0.001  (0.06) | 0.000  (0.04) | 0.000  (0.05) | 0.000  (0.02) | 0.000  (0.05) |
| anger | | -0.012  (-0.39) | -0.016  (-0.54) | 0.003  (0.13) | -0.023  (-0.76) | -0.009  (-0.27) | -0.021  (-0.68) | -0.010  (-0.32) | -0.007  (-0.20) | -0.014  (-0.44) | -0.012  (-0.37) | -0.021  (-0.69) | -0.007  (-0.22) |
| sad | | -0.013  (-0.41) | -0.010  (-0.32) | -0.011  (-0.36) | -0.010  (-0.32) | -0.020  (-0.68) | -0.019  (-0.64) | -0.016  (-0.54) | -0.016  (-0.52) | -0.017  (-0.56) | -0.017  (-0.58) | -0.017  (-0.55) | -0.019  (-0.61) |
| i | | -0.033  (-1.12) | -0.043  (-1.46) | -0.036  (-1.21) | -0.046  (-1.56) | -0.049  (-1.68) | -0.071  (-2.47) | -0.033  (-1.13) | -0.017  (-0.55) | -0.038  (-1.28) | -0.037  (-1.26) | -0.039  (-1.32) | -0.036  (-1.23) |
| you | | -0.029  (-0.99) | -0.037  (-1.26) | -0.035  (-1.21) | -0.035  (-1.18) | -0.047  (-1.62) | -0.042  (-1.45) | -0.035  (-1.21) | -0.034  (-1.16) | -0.036  (-1.22) | -0.037  (-1.24) | -0.038  (-1.29) | -0.043  (-1.48) |
| swear | | -0.032  (-1.07) | -0.008  (-0.23) | -0.015  (-0.50) | -0.023  (-0.76) | -0.015  (-0.50) | -0.028  (-0.94) | -0.015  (-0.50) | -0.021  (-0.71) | -0.023  (-0.76) | -0.014  (-0.46) | -0.026  (-0.89) | -0.019  (-0.62) |
| negate | | -0.025  (-0.84) | -0.033  (-1.14) | -0.026  (-0.87) | -0.029  (-0.97) | -0.032  (-1.09) | -0.039  (-1.32) | -0.022  (-0.73) | -0.023  (-0.75) | -0.023  (-0.77) | -0.022  (-0.73) | -0.031  (-1.05) | -0.029  (-0.99) |
| we | | -0.011  (-0.34) | -0.012  (-0.38) | -0.009  (-0.28) | -0.011  (-0.36) | -0.011  (-0.36) | -0.013  (-0.42) | -0.013  (-0.41) | -0.011  (-0.33) | -0.013  (-0.42) | -0.013  (-0.42) | -0.010  (-0.33) | -0.011  (-0.34) |
| ratio we | | -0.037  (-1.27) | -0.039  (-1.32) | -0.033  (-1.11) | -0.038  (-1.31) | -0.042  (-1.45) | -0.056  (-1.93) | -0.036  (-1.21) | -0.027  (-0.93) | -0.039  (-1.32) | -0.038  (-1.29) | -0.039  (-1.32) | -0.037  (-1.25) |
| article | | -0.029  (-0.99) | -0.026  (-0.86) | -0.027  (-0.91) | -0.034  (-1.16) | -0.031  (-1.06) | -0.048  (-1.63) | -0.023  (-0.79) | -0.018  (-0.61) | -0.029  (-0.97) | -0.027  (-0.90) | -0.039  (-1.34) | -0.028  (-0.93) |
| posemo | | -0.006  (-0.20) | -0.003  (-0.08) | -0.007  (-0.21) | -0.005  (-0.15) | 0.000  (0.03) | -0.001  (0.00) | -0.003  (-0.08) | -0.005  (-0.14) | -0.004  (-0.12) | -0.002  (-0.05) | -0.007  (-0.22 | -0.001  (-0.01) |

Note: Indicators of depression above the dashed line. Indicators of adjustment below the dashed line. Negemo: negative emotions; anx: anxiety; sad: sadness; i: first person singular pronouns, you: second person pronouns, swear: swear words; negate: negations; we: person plural pronouns; ratio we: ratio of first person plural pronouns over all first person pronouns; posemo: positive emotions; ADI: Area Deprivation Index; EHI: Economic hardship and inequality; EA: Educational attainment; FS: Financial strength; <9^th^ grade educ.: less than ninth grade education; >= HS educ.: at least highschool education; unempl.: unemployed; Med. Fam. Income: Median family income

Tables S2. Spatial lag regression Betas for percentage of different ethnic groups as independent variables (controlling for population density, proportion of males and median age)

|  | % Asian  (IV) | | | % Afro-American  (IV) | | | % Latino  (IV) | | | % White  (IV) | | |
| --- | --- | --- | --- | --- | --- | --- | --- | --- | --- | --- | --- | --- |
|  | direct | indirect | total | direct | indirect | total | direct | indirect | total | direct | indirect | total |
| negemo | -0.07 * | -0.03 * | -0.11 * | 0.13 *** | 0.06 *** | 0.19 *** | 0.23 *** | 0.09 *** | 0.32 *** | -0.14 *** | -0.07 *** | -0.21 *** |
| anx | 0.01 | 0 | 0.01 | -0.05 | 0 | -0.05 | 0.04 | 0 | 0.04 | 0.05 | 0 | 0.04 |
| anger | -0.05 | -0.02 | -0.07 | 0.22 *** | 0.11 *** | 0.33 *** | 0.26 *** | 0.12 *** | 0.38 *** | -0.25 *** | -0.12 *** | -0.37 *** |
| sad | -0.11 *** | -0.02 ** | -0.13 *** | -0.12 *** | -0.02 ** | -0.14 *** | 0.14 *** | 0.02 ** | 0.16 *** | 0.13 *** | 0.02 ** | 0.15 *** |
| i | -0.2 *** | -0.17 *** | -0.37 *** | 0.18 *** | 0.15 *** | 0.33 *** | 0.14 *** | 0.12 *** | 0.26 *** | -0.15 *** | -0.13 *** | -0.27 *** |
| you | -0.21 *** | -0.1 *** | -0.31 *** | 0.04 | 0.02 | 0.07 | 0 | 0 | 0.01 | 0.03 | 0.01 | 0.04 |
| swear | -0.04 | -0.03 | -0.07 | 0.37 *** | 0.23 *** | 0.6 *** | 0.24 *** | 0.16 *** | 0.41 *** | -0.4 *** | -0.23 *** | -0.63 *** |
| negate | -0.14 *** | -0.04 *** | -0.19 *** | 0.26 *** | 0.06 *** | 0.32 *** | 0.02 | 0.01 | 0.03 | -0.19 *** | -0.05 *** | -0.24 *** |
| we | -0.02 | 0 | -0.02 | -0.12 *** | -0.03 ** | -0.15 *** | -0.11 *** | -0.02 ** | -0.13 *** | 0.17 *** | 0.03 *** | 0.2 *** |
| ratio we | 0.12 *** | 0.07 *** | 0.18 *** | -0.18 *** | -0.1 *** | -0.28 *** | -0.14 *** | -0.08 *** | -0.21 *** | 0.19 *** | 0.1 *** | 0.29 *** |
| article | 0.15 *** | 0.07 *** | 0.22 *** | -0.35 *** | -0.12 *** | -0.47 *** | -0.19 *** | -0.07 *** | -0.26 *** | 0.33 *** | 0.12 *** | 0.45 *** |
| posemo | 0 | 0 | 0.01 | -0.14 *** | -0.03 ** | -0.17 *** | 0.03 | 0.01 | 0.04 | 0.15 *** | 0.03 ** | 0.18 *** |

Note: Indicators of depression above the dashed line. Indicators of adjustment below the dashed line. Negemo: negative emotions; anx: anxiety; sad: sadness; i: first person singular pronouns, you: second person pronouns, swear: swear words; negate: negations; we: person plural pronouns; ratio we: ratio of first person plural pronouns over all first person pronouns; posemo: positive emotions.

Table S3. Spatial lag regression Betas for the ADI and the ADI-3 dimensions as independent variables (controlling for population density, proportion of males and median age)

|  | ADI | | | ADI-3 EHI | | | ADI-3 EA | | | ADI-3 FS | | |
| --- | --- | --- | --- | --- | --- | --- | --- | --- | --- | --- | --- | --- |
|  | direct | indirect | total | direct | indirect | total | direct | indirect | total | direct | indirect | total |
| negemo | 0.19 *** | 0.09 *** | 0.28 *** | 0.18 *** | 0.08 *** | 0.26 *** | -0.24 *** | -0.09 *** | -0.34 *** | -0.13 *** | -0.06 *** | -0.2 *** |
| anx | -0.02 | 0 | -0.02 | -0.01 | 0 | -0.01 | 0 | 0 | 0 | 0.03 | 0 | 0.03 |
| anger | 0.19 *** | 0.1 *** | 0.29 *** | 0.19 *** | 0.1 *** | 0.28 *** | -0.28 *** | -0.12 *** | -0.4 *** | -0.11 *** | -0.06 *** | -0.17 *** |
| sad | 0.14 *** | 0.02 ** | 0.16 *** | 0.09 ** | 0.01 * | 0.1 ** | -0.11 *** | -0.02 ** | -0.13 *** | -0.15 *** | -0.02 ** | -0.17 *** |
| i | 0.36 *** | 0.25 *** | 0.6 *** | 0.3 *** | 0.23 *** | 0.54 *** | -0.27 *** | -0.19 *** | -0.46 *** | -0.32 *** | -0.25 *** | -0.57 *** |
| you | 0.2 *** | 0.09 *** | 0.29 *** | 0.08 ** | 0.04 * | 0.12 ** | -0.08 ** | -0.04 ** | -0.12 ** | -0.28 *** | -0.12 *** | -0.4 *** |
| swear | 0.22 *** | 0.17 *** | 0.39 *** | 0.24 *** | 0.18 *** | 0.42 *** | -0.3 *** | -0.19 *** | -0.49 *** | -0.12 *** | -0.1 *** | -0.22 *** |
| negate | 0.21 *** | 0.06 *** | 0.27 *** | 0.17 *** | 0.05 *** | 0.22 *** | -0.13 *** | -0.04 *** | -0.18 *** | -0.21 *** | -0.06 *** | -0.27 *** |
| we | -0.07 * | -0.01 * | -0.08 * | -0.09 ** | -0.02 * | -0.11 ** | 0.1 ** | 0.02 ** | 0.12 ** | 0.02 | 0 | 0.02 |
| ratio we | -0.28 *** | -0.14 *** | -0.42 *** | -0.26 *** | -0.13 *** | -0.39 *** | 0.22 *** | 0.11 *** | 0.33 *** | 0.24 *** | 0.13 *** | 0.37 *** |
| article | -0.32 *** | -0.11 *** | -0.43 *** | -0.28 *** | -0.11 *** | -0.38 *** | 0.32 *** | 0.11 *** | 0.42 *** | 0.25 *** | 0.1 *** | 0.35 *** |
| posemo | -0.11 *** | -0.02 ** | -0.13 *** | -0.2 *** | -0.04 *** | -0.23 *** | 0.07 * | 0.01 | 0.08 * | 0.04 | 0.01 | 0.04 |

Note: Indicators of depression above the dashed line. Indicators of adjustment below the dashed line. Negemo: negative emotions; anx: anxiety; sad: sadness; i: first person singular pronouns, you: second person pronouns, swear: swear words; negate: negations; we: person plural pronouns; ratio we: ratio of first person plural pronouns over all first person pronouns; posemo: positive emotions; ADI: Area Deprivation Index; EHI: Economic hardship and inequality; EA: Educational attainment; FS: Financial strength

Tables S4. Spatial lag regression Betas for percent of residents with less than ninth grade education, at least highschool education, unemployed as well as median family income as independent variables (controlling for population density, proportion of males and median age)

|  | % < 9th grade edu. | | | % >= HS edu. | | | % unemployed | | | Median fam. Income | | |
| --- | --- | --- | --- | --- | --- | --- | --- | --- | --- | --- | --- | --- |
|  | direct | indirect | total | direct | indirect | total | direct | indirect | total | direct | indirect | total |
| negemo | 0.2 *** | 0.08 *** | 0.29 *** | -0.25 *** | -0.1 *** | -0.35 *** | 0.25 *** | 0.1 *** | 0.36 *** | -0.15 *** | -0.07 *** | -0.22 *** |
| anx | 0 | 0 | 0 | 0.02 | 0 | 0.02 | 0.03 | 0 | 0.03 | 0.01 | 0 | 0.01 |
| anger | 0.25 *** | 0.11 *** | 0.36 *** | -0.29 *** | -0.12 *** | -0.41 *** | 0.27 *** | 0.13 *** | 0.4 *** | -0.14 *** | -0.07 *** | -0.21 *** |
| sad | 0.09 ** | 0.01 * | 0.1 ** | -0.12 *** | -0.02 ** | -0.13 *** | 0.08 ** | 0.01 * | 0.09 ** | -0.14 *** | -0.02 ** | -0.16 *** |
| i | 0.22 *** | 0.17 *** | 0.39 *** | -0.33 *** | -0.22 *** | -0.55 *** | 0.27 *** | 0.22 *** | 0.5 *** | -0.32 *** | -0.24 *** | -0.56 *** |
| you | 0.06 | 0.03 | 0.09 | -0.14 *** | -0.07 *** | -0.21 *** | 0.05 | 0.03 | 0.07 | -0.2 *** | -0.09 *** | -0.29 *** |
| swear | 0.25 *** | 0.17 *** | 0.42 *** | -0.32 *** | -0.2 *** | -0.51 *** | 0.32 *** | 0.22 *** | 0.54 *** | -0.17 *** | -0.13 *** | -0.29 *** |
| negate | 0.11 *** | 0.04 ** | 0.15 *** | -0.21 *** | -0.06 *** | -0.27 *** | 0.14 *** | 0.04 *** | 0.18 *** | -0.18 *** | -0.05 *** | -0.24 *** |
| we | -0.08 ** | -0.02 * | -0.1 ** | 0.1 *** | 0.02 ** | 0.13 *** | -0.13 *** | -0.03 ** | -0.16 *** | 0.04 | 0.01 | 0.04 |
| ratio we | -0.17 *** | -0.09 *** | -0.27 *** | 0.27 *** | 0.13 *** | 0.39 *** | -0.25 *** | -0.13 *** | -0.39 *** | 0.25 *** | 0.13 *** | 0.38 *** |
| article | -0.27 *** | -0.1 *** | -0.37 *** | 0.37 *** | 0.11 *** | 0.48 *** | -0.28 *** | -0.11 *** | -0.38 *** | 0.27 *** | 0.1 *** | 0.37 *** |
| posemo | -0.04 | -0.01 | -0.05 | 0.07 * | 0.02 * | 0.09 * | -0.17 *** | -0.03 *** | -0.2 *** | 0.1 ** | 0.02 ** | 0.12 ** |

Note: Indicators of depression above the dashed line. Indicators of adjustment below the dashed line. Negemo: negative emotions; anx: anxiety; sad: sadness; i: first person singular pronouns, you: second person pronouns, swear: swear words; negate: negations; we: person plural pronouns; ratio we: ratio of first person plural pronouns over all first person pronouns; posemo: positive emotions; <9^th^ grade educ.: less than ninth grade education; >= HS educ.: at least highschool education; unempl.: unemployed; Med. Fam. Income: Median family income

Table S5. Spatial lag regression Betas for the number of poor physical and mental health days regressed on the LIWC-2015 indicators of depression and adjustment.

|  | IVs | direct | indirect | total | Moran's I (deviate) |
| --- | --- | --- | --- | --- | --- |
| PoorPH (DV) | negemo | 0.15 *** | 0.13 *** | 0.28 *** | -0.021 (-0.69) |
|  | anx | -0.02 | -0.02 | -0.03 | -0.022 (-0.73) |
|  | anger | 0.13 *** | 0.12 *** | 0.25 *** | -0.017 (-0.56) |
|  | sad | 0.13 *** | 0.12 *** | 0.24 *** | -0.024 (-0.80) |
|  | i | 0.32 *** | 0.25 *** | 0.58 *** | -0.007 (-0.22) |
|  | you | 0.13 *** | 0.12 *** | 0.25 *** | -0.029 (-0.98) |
|  | swear | 0.13 *** | 0.12 *** | 0.24 *** | -0.012 (-0.40) |
|  | negate | 0.17 *** | 0.15 *** | 0.32 *** | -0.022 (-0.74) |
|  | we | -0.02 | -0.02 | -0.04 | -0.022 (-0.75) |
|  | ratio_we | -0.21 *** | -0.18 *** | -0.39 *** | -0.021 (-0.70) |
|  | article | -0.18 *** | -0.16 *** | -0.35 *** | -0.022 (-0.74) |
|  | posemo | -0.09 *** | -0.08 ** | -0.17 ** | -0.018 (-0.59) |
| PoorMH (DV) | negemo | 0.1 *** | 0.1 *** | 0.2 *** | -0.015 (-0.48) |
|  | anx | 0.01 | 0.01 | 0.02 | -0.015 (-0.51) |
|  | anger | 0.07 ** | 0.08 ** | 0.15 ** | -0.013 (-0.44) |
|  | sad | 0.1 *** | 0.1 *** | 0.2 *** | -0.016 (-0.53) |
|  | i | 0.25 *** | 0.24 *** | 0.49 *** | -0.009 (-0.29) |
|  | you | 0.1 *** | 0.1 *** | 0.19 *** | -0.020 (-0.67) |
|  | swear | 0.05 * | 0.05 | 0.11 * | -0.012 (-0.40) |
|  | negate | 0.12 *** | 0.12 *** | 0.23 *** | -0.017 (-0.55) |
|  | we | -0.01 | -0.01 | -0.02 | -0.015 (-0.51) |
|  | ratio_we | -0.17 *** | -0.16 *** | -0.33 *** | -0.018 (-0.58) |
|  | article | -0.12 *** | -0.12 *** | -0.24 *** | -0.015 (-0.50) |
|  | posemo | -0.08 ** | -0.08 ** | -0.15 ** | -0.012 (-0.40) |

Note: Indicators of depression above the dashed lines. Indicators of adjustment below the dashed lines. Negemo: negative emotions; anx: anxiety; sad: sadness; i: first person singular pronouns, you: second person pronouns, swear: swear words; negate: negations; we: person plural pronouns; ratio we: ratio of first person plural pronouns over all first person pronouns; posemo: positive emotions. PoorPH: Days with poor physical health. PoorMH: Days with poor mental health.


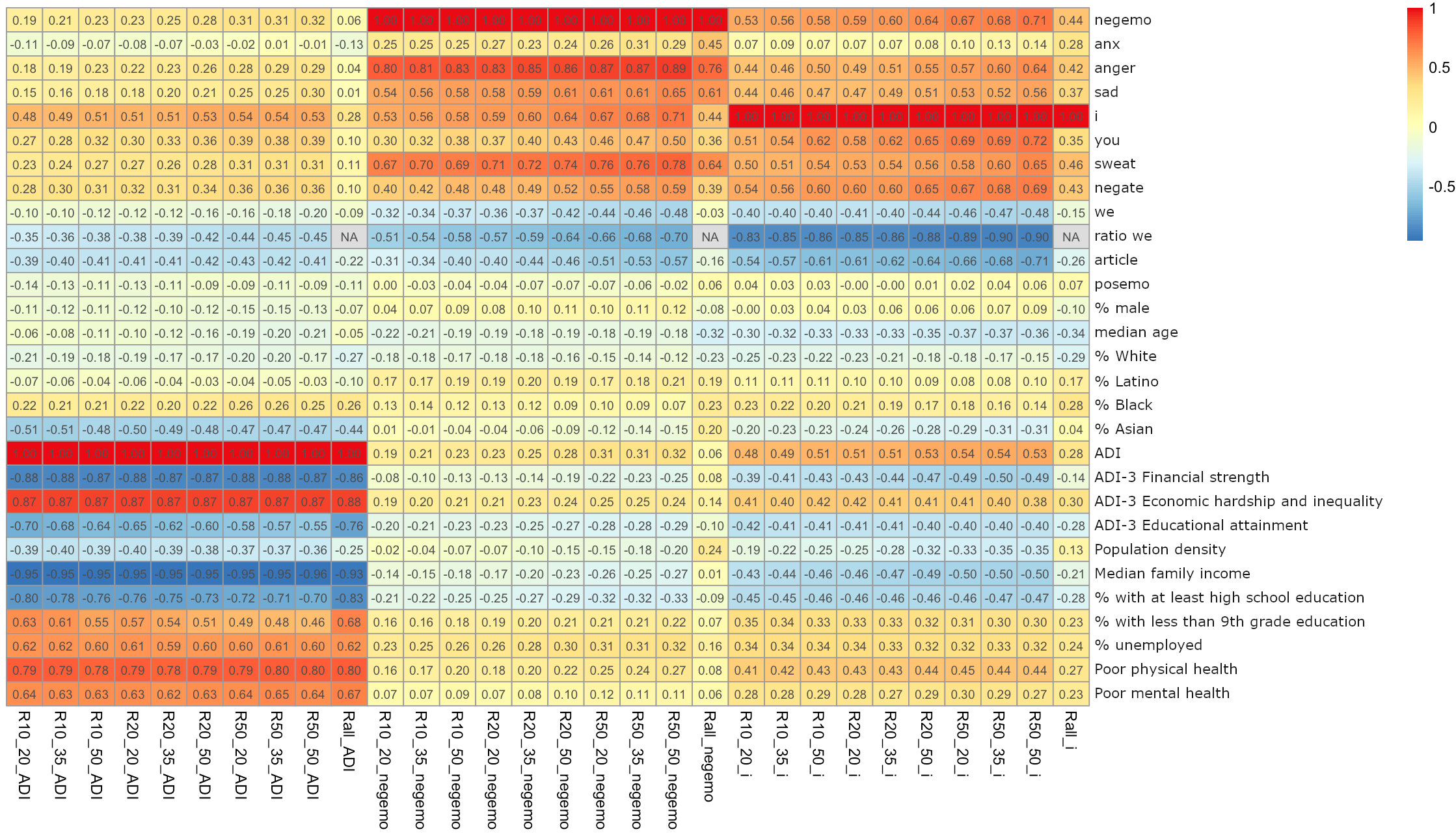


**Figure S1.** Zero-order correlations of the ADI, the LWIC 2015 negative emotions category, the LWIC 2015 first personal pronoun category with all study variables at with different county inclusion criteria. First number in the column name refers to the minimum number of tweets per day, second number refers to the minimum number of days during which the minimum number of tweets has been collected (e.g., R10_20: minimum 20 days with at least 10 tweets collected). Rall: all counties included. Negemo: negative emotions; anx: anxiety; sad: sadness; i: first person singular pronouns, you: second person pronouns, swear: swear words; negate: negations; we: person plural pronouns; ratio we: ratio of first person plural pronouns over all first person pronouns; posemo: positive emotions; ADI: Area Deprivation Index.
